# Probing pain aversion in rats with the “Heat Escape Threshold” paradigm

**DOI:** 10.1177/17448069231156657

**Published:** 2023-03-06

**Authors:** Roni Hogri, Bozhidar Baltov, Ruth Drdla-Schutting, Valeria Mussetto, Holzinger Raphael, Lidia Trofimova, Jürgen Sandkühler

**Affiliations:** Department of Neurophysiology, 31235 Center for Brain Research, Medical University of Vienna, Vienna, Austria

**Keywords:** Pain behaviour, heat hyperalgesia, aversion, escape, inflammatory pain, opioid analgesia, opioid withdrawal, lateral parabrachial nucleus

## Abstract

The aversive aspect of pain constitutes a major burden faced by pain patients. This has been recognized by the pain research community, leading to the development of novel methods focusing on affective-motivational behaviour in pain model animals. The most common tests used to assess pain aversion in animals require cognitive processes, such as associative learning, complicating the interpretation of results. To overcome this issue, studies in recent years have utilized unconditioned escape as a measure of aversion. However, the vast majority of these studies quantify jumping – a common escape behaviour in mice, but not in adult rats, thus limiting its use. Here, we present the “Heat Escape Threshold” (HET) paradigm for assessing heat aversion in rats. We demonstrate that this method can robustly and reproducibly detect the localized effects of an inflammatory pain model (intraplantar carrageenan) in male and female Sprague-Dawley rats. In males, a temperature that evoked unconditioned escape following carrageenan treatment also induced real-time place avoidance (RTPA). Systemic morphine more potently alleviated carrageenan-induced heat aversion (as measured by the HET and RTPA methods), as compared to reflexive responses to heat (as measured by the Hargreaves test), supporting previous findings. Next, we examined how blocking of excitatory transmission to the lateral parabrachial nucleus (LPBN), a key node in the ascending pain system, affects pain behaviour. Using the HET and Hargreaves tests, we show that intra-LPBN application of glutamate antagonists reverses the effects of carrageenan on both affective and reflexive pain behaviour, respectively. Finally, we employed the HET paradigm in a generalized opioid-withdrawal pain model. Withdrawal from a brief systemic administration of remifentanil resulted in a long-lasting and robust increase in heat aversion, but no change in reflexive responses to heat. Taken together, these data demonstrate the utility of the HET paradigm as a novel tool in preclinical pain research.

## Introduction

Pain is a multi-dimensional experience, involving sensorimotor, emotional, and cognitive aspects.^1^ For many decades, the study of pain behaviour in animal models has focused almost exclusively on “reflexive” nocifensive responses such as paw withdrawal in response to mechanical pressure and heat (the von Frey and Hargreaves tests, respectively).^2,3^ While these methods have been invaluable for advancing our understanding of pain mechanisms, they do not address the aversive aspect of pain, which arguably constitutes the major burden faced by chronic pain patients, many of whom suffer from psychological co-morbidities such as anxiety, depression, and social withdrawal.^4–7^ Indeed, the clinical relevance of studies involving discrete reflexive responses has been questioned, and suggested as an explanation for translational failures.^2,3,8–10^ This realization has led to a substantial increase in studies looking into emotional-motivational processing in animal pain models, predominantly in rodents.^2,10,11^

In rodents, pain aversion is commonly assessed via aversive learning paradigms. For example, the conditioned place aversion/preference (CPA/CPP) paradigm has been extensively used to assess the aversiveness of pain, as well as the effectiveness of analgesic or anti-aversive interventions.^12–19^ This paradigm requires that animals learn to associate a specific location (typically one compartment of a behavioural arena) with the presence of pain, or its relief. Effective learning often requires multiple conditioning sessions over several days, and the interpretation of results is complicated by possible effects of interventions (e.g., analgesic drugs) on learning and memory. Another set of learning paradigms involves the establishment of real-time place avoidance (RTPA). In such paradigms, the avoidance of the area associated with pain can typically be achieved within a single session,^20–23^ minimizing issues related to memory consolidation. However, this paradigm requires that peripheral somatosensory stimulation (e.g., mechanical pressure applied with a von Frey filament) is experienced as painful when applied to an affected area of the body (e.g., an inflamed hindpaw), but not when applied to an unaffected area (e.g., the contralateral hindpaw). This limitation impedes the use of the RTPA paradigm for the assessment of pain aversion in animal models of generalized pain, such as those induced by chemotherapeutic drugs or opioid withdrawal.^10,24–26^

The need for assessing unconditioned emotional-motivational pain behaviours has led to the development of paradigms in which escape behaviour serves as a proxy for aversion.^17,23,27,28^ Such experiments typically involve counting the number of jumps in mice subjected to nociceptive input. Importantly, this form of escape is species-specific, and is not observed in adult rats, which are frequently used to study the neurobiology of pain and its resultant behaviour.

In the current study, we developed the “Heat Escape Threshold” (HET) paradigm as a method for measuring heat-evoked escape responses in rats. We show that the HET paradigm can reliably and robustly detect heat aversion in a localized inflammatory pain model (intraplantar carrageenan). Further, we report that systemic morphine was more potent in reversing carrageenan effects on heat aversion (as tested with the HET and RTPA paradigms) as compared to carrageenan’s effects on reflexive behaviour (as assessed with the Hargreaves test). Blocking glutamatergic inputs to the LPBN – a key node in the ascending pain system, abolished carrageenan-induced changes to both reflexive and aversive responses to heat, as measured by the HET and Hargreaves methods, respectively. Finally, we demonstrate that the HET paradigm can detect heat aversion in an opioid withdrawal model that does not affect reflexive responses to heat. These results demonstrate the usefulness of the HET paradigm in addressing fundamental questions in pain research.

## Materials and methods

### Animals

All experiments were performed on Sprague-Dawley rats (55–90 days old), obtained from the Medical University of Vienna breeding facility (Himberg, Austria), or from Janvier Laboratories (Saint Berthevin Cedex, France). Experiments were performed on male subjects, unless stated otherwise. All experimental procedures were approved by the Austrian Federal Ministry of Education, Science and Research (BMBWF), and were performed according to European Communities Council directives on the use of animals for scientific purposes (2010/63/EU). Animals were housed in a controlled environment (22 ± 2°C, 55 ± 10% humidity, 12 h light/dark cycle), with *ad libitum* access to food and water. Behavioural testing was performed during the light phase, in designated rooms (light intensity = 60–80 lux).

### General experimental procedures

Pain behaviour was measured in multiple daily sessions. Unless stated otherwise, one baseline session (day -1) was performed before pain model induction (or control procedure). One day later (day 0), a pain model (inflammatory pain or opioid withdrawal) was induced in a subset of animals; subsequently, a test session, identical to the baseline session, took place. For the Hargreaves test, two baseline sessions were performed (days -2 and -1), and the withdrawal latencies per paw across these two days were averaged. In experiments involving opioid withdrawal, the baseline session (day -1) was followed by multiple test sessions (days 0, 1, 3, and 7). In all experiments, the experimenter performing the behavioural experiments was blinded to the group allocation of each rat.

### Intraplantar carrageenan model of inflammatory pain

Animals were placed in an anaesthesia induction chamber, and received isoflurane anaesthesia (5% in 6 L/min O_2_) until loss of reflexive responses to hindpaw pinch (∼90 s). For induction of inflammatory pain, an intraplantar injection of carrageenan (0.5% w/v in 0.1 mL 0.9% NaCl) was delivered to the left hindpaw, using a 24G needle. Control rats were anaesthetised as described above, but not injected, in order to avoid inflammation related to mechanical injury. Behavioural testing recommenced 3 h after recovery from anaesthesia.

### Remifentanil withdrawal model

Rats were placed in an anaesthesia induction chamber and received isoflurane anaesthesia (4% in a 1:1 N_2_O/O_2_ mixture). After loss of consciousness, rats were intubated with a 16G cannula and were mechanically ventilated (75 strokes/min, tidal volume of 4–6 mL). Subsequently, anaesthesia was maintained using 1.5% isoflurane. A deep level of anaesthesia was verified by lack of withdrawal reflex to hindpaw pinch. Surgery was performed using sterilized tools and materials. The jugular vein was exposed and cannulated, and remifentanil (Ultiva; GlaxoSmithKline, Vienna, Austria), dissolved in 0.9% NaCl, was given as a bolus injection (30 μg/kg) followed by a 1 h infusion at a rate of 450 μg/kg/h. Control rats were injected with 0.9% NaCl. Next, cannulation was removed and the skin was sutured; 15 min later, anaesthesia and mechanical ventilation were discontinued, and rats were extubated. Behavioural testing recommenced 4 h after recovery from anaesthesia.

### Punctate heat probe

**Figure 1.**
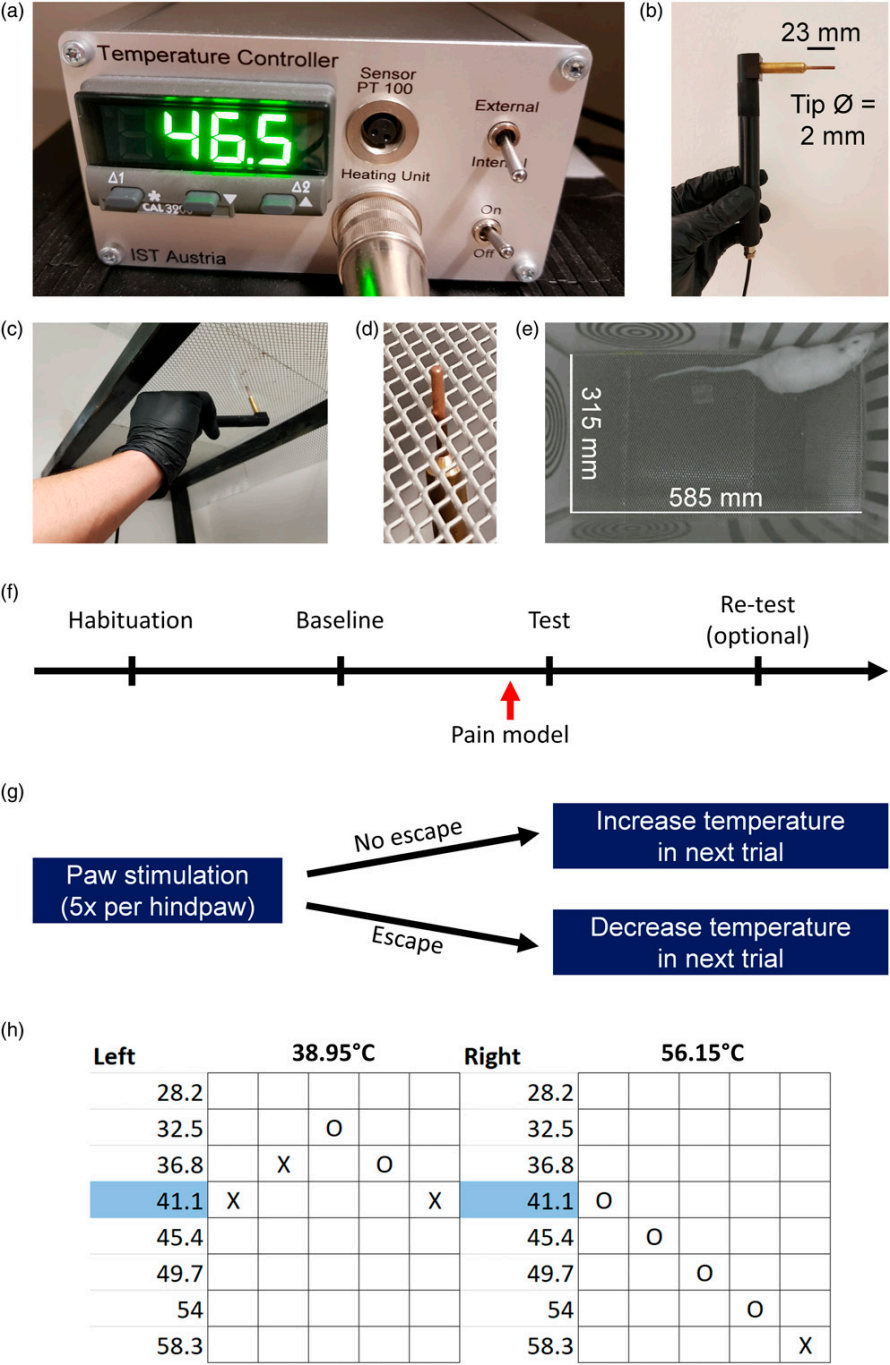
Experimental procedures. (a–b) A digital temperature controller (IST, Austria) controlled the temperature of a hand-held punctate heat probe. (c–e) Rats were placed in a behavioural arena (wall height = 280 mm) and received punctate heat stimulation through a mesh floor with rhomboid openings (each with an area of = 3 × 6 mm^2^). (f) Experimental design for the HET paradigm. Rats were habituated to the behavioural laboratory and experimenter for at least 1 h, followed by a baseline session. One day after the baseline session, a pain model was induced, and an additional measurement of escape thresholds was performed (test session). In some cases, additional re-test sessions (identical to the baseline and test sessions) were performed. (g) Experimental procedure for baseline and test sessions of the "Heat Escape Threshold" (HET) paradigm, using the simplified up-down (SUDO) design. The first stimulation was delivered 5 min after placement of the rat in the arena. Based on the response in the previous trial, the temperature was increased or decreased by 4.3°C. (h) Example of HET scoring in a rat with heat hypersensitivity in the left, but not right, hindpaw. X: Escape, O: No escape. For each hindpaw, the calculated 50% heat escape threshold is shown on top of the table, in bold.

The punctate heat probe was designed and produced in collaboration with the Miba Machine Shop, Institute of Science and Technology (IST), Austria. The device consists of a control unit (Figure 1(a)), which can be used to heat a copper rod (Figure 1(b)) to the desired temperature. The base of the copper rod contains a temperature sensor, providing feedback to the heat control unit. Due to the difference in the diameter of the base and the tip of the copper rod, there was a constant difference between the recorded temperature and the actual temperature at the tip of the copper rod. Therefore, the tip temperature was periodically recorded using an external temperature sensor (GTF 601, Greisinger, Germany) connected to a digital thermometer (GMH 3750, Greisinger). Measurements were digitized (PowerLab, AD Instruments), and compared to the set temperature shown on the control unit (in the range of 30–70°C, 5°C steps). These measurements confirmed that the difference between the set and tip temperatures were constant over time, such that: tip temperature = set temperature × 0.86 + 2.4°C. All temperature values reported henceforth refer to the tip temperature. The heat probe was applied to the hindpaws of rats through an elevated metal mesh (Figures 1(c) and (d)). A behavioural arena was mounted on top of the mesh (Figure 1(e)). For each experiment, a single rat was placed in the middle of the arena and allowed to move freely within it.

### HET paradigm

The time course of the HET paradigm is shown in Figure 1(f). In the habituation session, rats were placed in the experimental room for at least 1 h and shortly handled by the experimenter (5 min), but were not exposed to the experimental apparatus. Habituation was followed by a baseline session (at least 4 h after habituation, but typically on the next day), and then by a test session (one day after the baseline session). In both baseline and test sessions, the temperature threshold required to elicit an escape (see below) was determined. Baseline and test sessions were identical, except that the test session was preceded by the induction of a pain model (or control procedure). In some cases, multiple daily test sessions were performed. In each session, the 50% HET was determined using an adaptation of the simplified up-down (SUDO) method, previously developed for the von Frey test.^29^ The procedure for baseline and test sessions are illustrated in Figure 1(g). Rats were placed in the behavioural arena, and allowed to freely explore. After 5 min, stimulation with the heat probe began; the starting temperature was 41.1°C, unless stated otherwise. Each hindpaw was stimulated 5 times, in alternating trials separated by at least 2.5 min (i.e., at least 5 min interval between two stimulations of the same hindpaw). Hindpaw stimulation consisted of gently touching the plantar surface with the tip of the heat probe, for a maximum duration of 5 s. If stimulation of a given paw induced an escape response, the temperature for the next trial was reduced by 4.3°C; if no escape was observed, the temperature for the next trial was increased by 4.3°C. Stimulation was only performed when rats were stationary and when all paws were in contact with the mesh floor. An escape response was defined as a movement of all four paws in response to hindpaw stimulation. To exclude escape responses that were triggered by experimenter approach or other environmental stimuli, the probe had to be in contact with the hindpaw for at least 1 s for an escape response to be considered valid. If a trial was considered invalid, it was repeated once the animal was stationary again. If a discrete withdrawal of the stimulated hindpaw was observed, stimulation was ceased and the trial was recorded as no escape. For each hindpaw, the 50% HET was calculated as the temperature used for the last stimulation ± half of one temperature step (4.3°C), depending on whether or not escape was induced in the last trial (−2.15°C if escape was induced, +2.15°C if not). A scoring example is shown in Figure 1(h). The starting temperature (41.1°C) and step intervals (4.3°C) were determined based on pilot experiments in carrageenan-treated rats, taking the following considerations into account: i. for carrageenan-treated paws, the mean 50% HET should be within ∼1 step of the starting temperature; ii. response thresholds should be normally distributed around the mean 50% HET; iii. the highest temperature applied in a series of 5 stimuli should be sufficient to evoke responses from the non-affected paw in the majority of cases. In one control experiment, the starting temperature was set to 23.9°C, and stimuli were delivered until at least one escape response per paw was observed.

### Real-time place avoidance paradigm

To confirm the aversiveness of punctate heat stimulation of the hindpaw in inflammatory pain model rats, a RTPA paradigm was used. The experimental setup was kept as similar as possible to that of the HET. Importantly, the same behavioural arena and heat probe were used, as well as a similar time course, consisting of 3 daily sessions (habituation, baseline, and test). The arena was divided into two “compartments”, that were distinguishable by markings on the walls (Figure 1(e)) as well as odorants used to clean the mesh floor (30% ethanol or 1% acetic acid). There was no physical barrier between compartments. Rats were placed in the middle of the behavioural arena, and allowed to explore freely. For each session, the compartment that was occupied at the end of the first 5 min was designated “the left paw compartment”. During the next 15 min, the punctate heat probe, set to 45.4°C, was used to stimulate either the left or right hindpaw with 30 s inter-stimulus intervals, depending on the location of the animal (left/right paw compartment). Each stimulus was delivered for a maximum of 5 s, or until withdrawal or escape were observed. The percent of time spent in the left paw compartment during the last 10 min of each session is reported. When testing the effects of systemic morphine in the RTPA paradigm (see below), locomotion was also assessed by calculating the number of crossings between the two compartments per minute during the last 10 min of each session.

### Hargreaves test

The Hargreaves test was used to assess nocifensive withdrawal responses to radiant heat, and was performed as previously described.^30–32^ Briefly, rats were placed on a glass surface, and an infrared light source (Stoelting, USA) was used to generate a radiant heat beam (150 mW/cm^2^/s; I.R. Heat-Flux Radiometer, Ugo Basile S.R.L., Italy) that was directed at the plantar surface of either the left or right hindpaw (alternating trials, 5 min inter-trial interval), for a maximum of 20 s per trial. In each session, each paw was stimulated 3 times, and the mean withdrawal latency was determined.

### Systemic morphine injections

In one set of experiments, the dose-dependent effects of systemic morphine on different pain behaviours were examined in carrageenan-treated rats. Morphine hydrochloride (Vendal, Gerot-Lannach, Austria) was diluted in 0.9% NaCl and loaded into 1 mL syringes. Each rat received a single i.p. injection (1 mL/kg) of either morphine (0.5, 1, or 3 mg/kg), or 0.9% NaCl as a vehicle control, 30 min before testing (2.5 h after intraplantar carrageenan injection), and was included in one behavioural test.

### Microinjections into the lateral parabrachial nucleus

In one experiment, the test session was performed after intra-LPBN microinjections. For this, rats were deeply anaesthetized using a combination of ketamine/xylazine cocktail (50 mg/kg and 5 mg/kg, respectively, i. p.) and isoflurane (1–3% in 1 L/min, via a face mask mounted on the stereotaxic apparatus). The head was shaved and fixed in a stereotaxic frame using non-rupturing ear bars. The scalp was cleaned with 70% ethanol, incised with a scalpel and retracted to expose the skull. Bur holes were drilled above the LPBN bilaterally (2.2 mm laterally to the lambda), and 26G stainless steel guide cannulae (C315 G, Plastics One, USA) were advanced at a 7° rostrocaudal angle, to a depth of 4.1 mm; dummy cannulae (C315DC, Plastics One) were used to prevent blockage. A combination of UV light-sensitive cement (Tetric *EvoFlow*, Ivoclar Vivadent) and bone cement (Refobacin, Biomet) was used to fix guide cannulae to four surgical screws (Ø1.2 mm; Precision Technology Supplies Ltd, UK) embedded in the skull. Throughout the surgery, a deep level of anaesthesia was verified by lack of reflexive responses to hindpaw pinch. Rats were allowed to recover for at least one week before behavioural procedures began. To perform intra-LPBN microinjections, the dummy cannula was replaced with a 33G injection cannula (C315I, Plastics One), which projected 2 mm from the guide. A polyethylene tube was used to connect the injection cannula to a microsyringe (10 μL, Hamilton, USA), mounted on a pump (LEGATO 100, KD Scientific, USA). In each animal, 500 nL of either a cocktail consisting of 6-cyano-7-nitroquinoxaline-2,3-dione (CNQX; Abcam, UK; 3.3 mM) and D-(-)-2-Amino-5-phosphonopentanoic acid (AP5; Tocris, UK; 34 mM),^33^ or 0.9% NaCl as a vehicle control, were bilaterally injected into the LPBN, at a rate of 5.55 nL/s. To mark injection locations, the injectant contained the fluorescent dye 1,1′-Dioctadecyl-3,3,3′,3′-Tetramethylindodicarbocyanine, 4-Chlorobenzenesulfonate Salt (DiD; Invitrogen, USA; 0.25% w/v). At the end of the experiment, rats were killed with an overdose of pentobarbital sodium (Exagon, Richter Pharma), and were transcardially perfused with heparinized 0.9% NaCl followed by 4% paraformaldehyde (PFA). Brains were extracted and kept overnight in 4% PFA, then cryoprotected in 20% and 30% sucrose in 0.1 M phosphate buffer (24 h each), flash-frozen and stored in −80°C until further processing. Coronal brainstem slices (40 μm) were prepared with a cryostat (CM3050S, Leica Microsystems, Germany) and mounted on microscope slides. A fluorescence microscope (BX51, Olympus, Japan) was used to determine microinjection locations; only animals in which DiD traces could be observed within the LPBN bilaterally were included in subsequent analyses.

### Statistical analysis

Each experiment was performed on a separate cohort of rats. For experiments examining carrageenan effects using the Hargreaves and HET methods, data were analysed separately for the left (carrageenan-treated) and right (control) hindpaw. For experiments involving the remifentanil withdrawal pain model, threshold values from both hindpaws of each rat were averaged, since this model was previously shown to induce generalized mechanical hypersensitivity.^24^ Statistical analyses were performed with GraphPad Prism (GraphPad Software, USA). For all experiments, a two-way ANOVA was used to determine the significance of the session × treatment interaction. Significant interactions were followed-up by Sidak’s multiple comparisons tests, which were used to detect significant differences between baseline and test sessions, and between conditions during the test session. In experiments comparing the efficacy of morphine doses in alleviating the effects of carrageenan treatment, the carrageenan effect was calculated as the difference between baseline and test sessions for the carrageenan-treated hindpaw. Effects sizes were calculated for the vehicle, 1 mg/kg, and 3 mg/kg groups; since the 0.5 mg/kg group was similar to the vehicle group in all tests (see Results), it was not included in this analysis. A one-way ANOVA, followed by Sidak’s multiple comparisons test, was used to compare between effect sizes for each test (Hargreaves, RTPA, and HET) separately. All data are reported as mean ± SEM, and *p* values <0.05 are considered statistically significant.

## Results

### HETs are robustly reduced in response to peripheral inflammation

**Figure 2.**
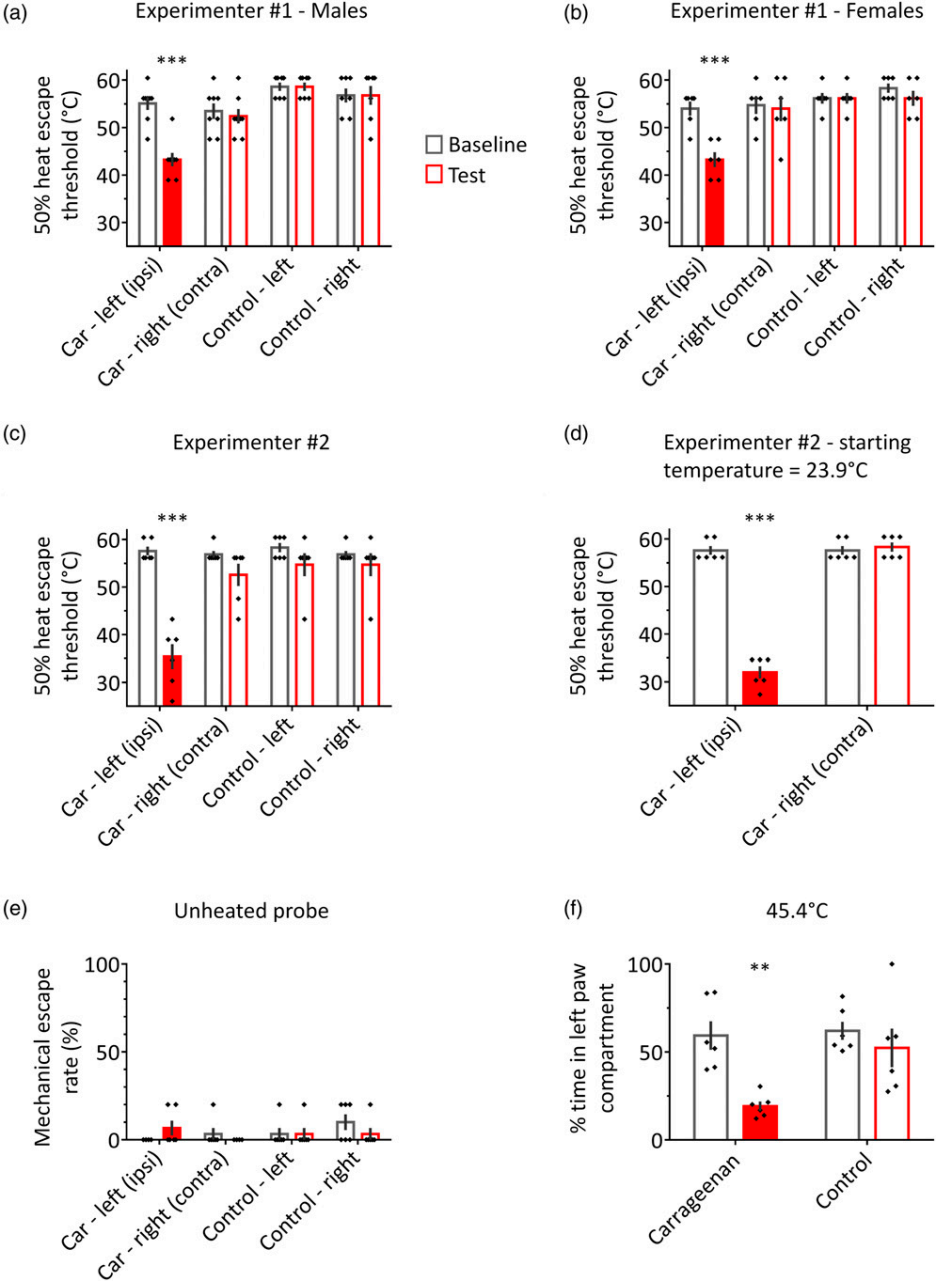
Effects of the intraplantar carrageenan inflammatory pain model on responses to heat probe stimulation. (a) In carrageenan-treated (car) male rats, escape thresholds to punctate heat stimulation were significantly reduced for the injected hindpaw, but not for the contralateral hindpaw. Car: *n* = 8, control: *n* = 7. (b) As in males, carrageenan treatment in female rats resulted in reduced escape thresholds for the injected hindpaw. *n* = 6 per group. (c) The experiment from (a) was replicated in a separate cohort of male rats, who were tested by a different experimenter. *n* = 6 per group. (d) In an additional cohort of carrageenan-injected rats tested by experimenter #2, the starting temperature was set to 23.9°C (compared to 41.1°C in a–c). *n* = 6. (e) When the punctate probe was not heated, escape behaviour was rarely observed (<4% of trials), and was not affected by paw inflammation. *n* = 6 per group. (f) In the real-time place avoidance paradigm, rats avoided the compartment in which the left hindpaw was stimulated with a temperature of 45.4°C following intraplantar carrageenan injection. *n* = 6 per group. **p < 0.01, ***p < 0.0001 compared to the baseline session, and compared to control measurements in the test session. “Ipsi” and “contra” refer to carrageenan-treated and control paws in car rats, respectively. Filled red bars indicate carrageenan treatment.

Intraplantar carrageenan is a commonly used inflammatory pain model, which induces a localized hypersensitivity to heat in the treated paw.^30,32^ Therefore, we first set out to examine whether carrageenan-induced heat hypersensitivity could be robustly detected using the HET paradigm. In male rats (Figure 2(a)), a two-way ANOVA revealed a significant session × treatment interaction (F (3, 26) = 14.5, *p* < 0.0001), with the 50% HET being significantly lower in carrageenan-treated hindpaws in the test session (43.3 ± 1.4°C) compared to the baseline session (55.1 ± 1.3°C; *p* < 0.0001), and compared to untreated hindpaws in the test session (55.9 ± 1.5°C, on average; *p* < 0.0001). No differences between the baseline and test sessions were observed in the contralateral hindpaw of carrageenan-treated rats, or in control rats (*p* > 0.92).

Similar results were observed in female rats (Figure 2(b)). The 50% HET of carrageenan-treated paws was significantly lower in the test session as compared to baseline (43.3 ± 1.6°C and 54 ± 1.5°C, respectively), and compared to untreated hindpaws in the test session (55.4 ± 1.8°C; ANOVA: *F* (3, 20) = 7.4, *p* = 0.002; Sidak multiple comparisons tests: *p* < 0.0001). No differences between baseline and test sessions were detected for untreated hindpaws (*p* > 0.7).

To examine the reliability of the HET paradigm, the experiment in male rats was replicated by an additional experimenter (Figure 2(c)). Again, the 50% HET of carrageenan-treated paws was lower in the test session (35.4 ± 2.6°C) compared to the baseline session (57.6 ± 0.9°C), and compared to untreated hindpaws in the test session (54 ± 2.4°C; ANOVA: *F* (3, 20) = 12.9, *p* < 0.0001; Sidak tests: *p* < 0.0001). There was no significant difference between baseline and test sessions for untreated hindpaws (*p* > 0.39). Taken together, these results show that the HET paradigm can robustly and reproducibly detect inflammation-induced localized hypersensitivity to heat in both male and female rats.

### HETs do not reflect aversive learning or mechanical hypersensitivity

In some cases, the 50% HET of carrageenan-treated paws was lower than the starting temperature of 41.1°C. Conceivably, application of painful heat may result in aversive learning that would promote escape behaviour in response to lower temperatures that would otherwise not induce escape. To examine this possibility, a cohort of carrageenan-treated rats was tested in a modified HET paradigm (Figure 2(d)), in which the starting temperature was 23.9°C – well below the escape thresholds observed in previous experiments. Carrageenan treatment caused a significant reduction in the 50% HET (ANOVA: *F* (3, 20) = 12.9, *p* < 0.0001; baseline: 57.6 ± 0.9°C; test: 32 ± 1.3°C; *p* < 0.0001); there was no significant difference between baseline and test sessions for the contralateral hindpaw (*p* = 0.9). Thus, similar results were obtained with the two different starting temperatures (compare Figures 2(c) and (d)), suggesting that the lowered 50% HET observed in carrageenan-treated paws was not caused by aversive learning.

Additionally to heat hypersensitivity, carrageenan treatment has previously been shown to induce hypersensitivity to mechanical pressure, as applied, for example, with von Frey filaments.^10,32^ In the HET paradigm, the heat probe makes contact with the hindpaw. To minimise the mechanical impact on HET results, the heat probe was gently applied to an area of the paw ∼25 times larger than that normally stimulated by von Frey filaments in rats, resulting in considerably lower pressure applied to the paw in the HET test. Nonetheless, the enhanced escape responses in carrageenan-treated rats could conceivably be explained by the mechanical aspect of the stimulation. To experimentally control for this possibility, we examined the extent to which escape responses could be evoked by stimulation of carrageenan-treated and control paws with an unheated probe (Figure 2(e)). In each rat, both hindpaws were stimulated over two sessions (baseline and test; 5 stimulations per session), and the percent of stimuli evoking an escape response was calculated per hindpaw per session. Overall, stimulation with the unheated probe evoked escape responses in <4% of trials. Moreover, the session × treatment interaction was not statistically significant (*F* (3, 20) = 2.8, *p* = 0.07), indicating that, in the absence of heat stimulation, escape responses were evoked to a similar degree in carrageenan-treated and control hindpaws.

Taken together, these results demonstrate that the HET paradigm measures heat-evoked unconditioned escape.

### Escape-evoking heat stimulation also induces real-time place avoidance

Escape behaviour in response to noxious stimuli has been suggested to reflect pain aversion.^17,23,27,28^ To further test the assumption that rats experience escape-evoking heat probe stimulation as aversive, the RTPA paradigm was employed (Figure 2(f)). For this, the heat probe was set to 45.4°C, a temperature that robustly evoked escape responses when applied to carrageenan-treated paws but not when applied to untreated paws. Depending on the arena compartment occupied by the rat, the probe was applied to either the right or left hindpaw. A two-way ANOVA revealed a significant session × treatment interaction (*F* (1, 10) = 5.8, *p* = 0.036), with carrageenan-treated rats spending less time in the left paw compartment in the test session (19.2 ± 2.7%) compared to the baseline session (59.4 ± 8%; *p* = 0.002), and compared to control rats in the test session (*p* < 0.01). Control rats spent a similar amount of time in the left paw compartment in both sessions (baseline: 62 ± 5.1%; test: 52.4 ± 11%; *p* = 0.51). These results confirm that rats experienced escape-evoking heat probe stimulation as aversive.

### Systemic morphine differentially affects carrageenan-induced pain aversion and reflexive responses

Previous studies have reported that opioids affect the aversive aspect of pain more potently than its sensory aspect.^13,16,18,34,35^ Therefore, we predicted that systemic morphine would more potently alleviate the effects of intraplantar carrageenan in the HET and RTPA paradigms as compared to the Hargreaves test, a well-established method for the measurement of discrete heat-evoked nocifensive responses (paw withdrawal). One day following baseline measurements, all rats received carrageenan injection into the left hindpaw, as well as a systemic (i.p.) injection of one of three doses of morphine (0.5, 1, or 3 mg/kg), or vehicle as control, and were tested in one of the three paradigms.

**Figure 3.**
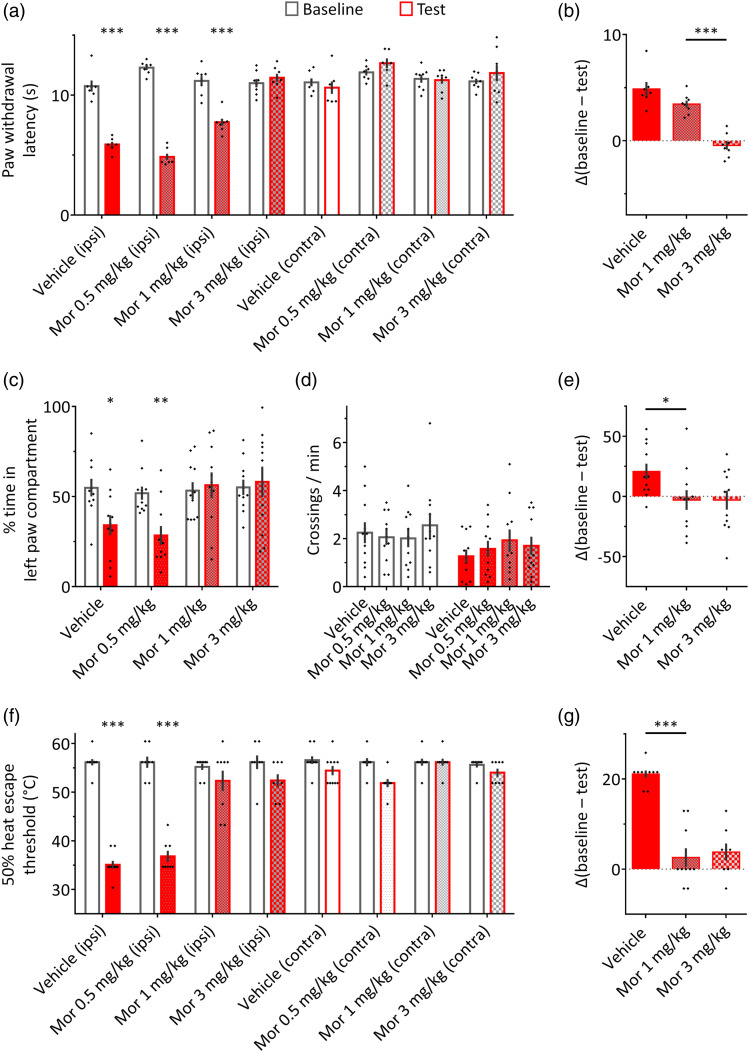
Differential effects of systemic morphine on reflexive and aversive responses to heat in rats with unilateral hindpaw inflammation. (a) In the Hargreaves test, withdrawal latencies were reduced for the carrageenan-injected hindpaw in rats receiving i.p. injections of vehicle (*n* = 7), 0.5 mg/kg morphine (*n* = 7), and 1 mg/kg morphine (*n* = 8), but not 3 mg/kg morphine (*n* = 8). (b) For each animal, the effect of carrageenan in the Hargreaves test was calculated as the difference in withdrawal latency of the carrageenan-treated paw between the baseline and test sessions (Δ). There was no significant difference between the vehicle and 1 mg/kg morphine groups. In contrast, the carrageenan effect was completely abolished by 3 mg/kg morphine. (c) In the real-time place avoidance paradigm (*n* = 11 per group), rats treated with vehicle or 0.5 mg/kg morphine showed a significant reduction in the time spent in the left paw compartment compared to baseline. No differences between baseline and test sessions were detected in rats treated with 1 or 3 mg/kg morphine. (d) Morphine treatment did not affect the number of crossings per minute in the RTPA paradigm, indicating that the prevention of place avoidance in rats treated with 1 and 3 mg/kg morphine was not related to an overall decrease in locomotion. (e) For each rat, the effect of carrageenan in the RTPA paradigm was calculated as the difference in time spent in the left paw stimulation compartment between the baseline and test sessions. Rats receiving 1 mg/kg morphine showed a significantly reduced carrageenan effect compared to vehicle-treated rats. (f) In the HET paradigm, escape thresholds were reduced for the carrageenan-treated hindpaws of rats receiving i.p. injections of vehicle (*n* = 10), or 0.5 mg/kg morphine (*n* = 8), but not 1 mg/kg (*n* = 9) or 3 mg/kg (*n* = 8) morphine. (g) For each animal, the effect of carrageenan in the HET paradigm was calculated as the difference in escape thresholds of the carrageenan-treated hindpaw between the baseline and test sessions. Rats receiving 1 mg/kg morphine showed a significantly reduced carrageenan effect compared to vehicle-treated rats; no difference was observed between rats injected with 1 mg/kg and 3 mg/kg morphine. *p < 0.05, **p < 0.01, ***p < 0.0001 (a, c, f: compared to the baseline session, and compared to the control hindpaw in the test session for the Hargreaves and HET tests; b, e, g: When comparing vehicle and 1 mg/kg morphine, or 1 mg/kg morphine and 3 mg/kg morphine). “Ipsi” and “contra” refer to carrageenan-treated and control hindpaws, respectively. Filled red bars indicate carrageenan treatment, and grey patterns indicate morphine treatment.

In the Hargreaves test, a reduction in paw withdrawal latencies following intraplantar carrageenan was prevented in rats treated with 3 mg/kg morphine (*p* > 0.95), but not in rats receiving 0.5 or 1 mg/kg morphine, or vehicle injection (session × treatment: *F* (7, 52) = 41.89, *p* < 0.0001; Sidak tests: *p* < 0.0001; Figure 3(a)). We then compared the size of the carrageenan effect (baseline – test paw withdrawal latencies of the carrageenan-treated paw) in the vehicle, 1 mg/kg morphine, and 3 mg/kg morphine groups (Figure 3(b)). This analysis showed that the carrageenan effect was abolished by 3 mg/kg as compared to 1 mg/kg morphine, but was not significantly affected by 1 mg/kg morphine as compared to vehicle (one-way ANOVA: *F* (3, 26) = 58.1, *p* < 0.0001; 3 mg/kg vs 1 mg/kg: *p* < 0.0001; 1 mg/kg versus vehicle: *p* = 0.057).

A higher efficacy of morphine was observed in the RTPA paradigm. Carrageenan-induced place avoidance was intact in rats receiving i.p. injections of vehicle or 0.5 mg/kg morphine (session x treatment: *F* (3, 40) = 4.1, *p* = 0.013; vehicle: *p* = 0.028; 0.5 mg/kg: *p* < 0.01), but was absent in rats receiving 1 and 3 mg/kg morphine (*p* > 0.98; Figure 3(c)). Morphine treatment did not affect locomotion, as indicated by the number of crossings between compartments (session x treatment: *F* (3,40) = 1.04, *p* = 0.39; Figure 3(d)). Analysis of carrageenan effect sizes showed that the carrageenan effect was significantly lower in the 1 mg/kg group compared to the vehicle group, but did not differ between the 1 and 3 mg/kg groups (one-way ANOVA: F (3, 40) = 4.1, *p* = 0.013; 1 mg/kg versus vehicle: *p* < 0.05; 3 mg/kg vs 1 mg/kg: *p* > 0.99; Figure 3(e)).

The effects of morphine in the HET paradigm were similar to those observed in the RTPA experiment. Carrageenan effectively reduced the 50% HET in rats receiving i.p. injections of vehicle or 0.5 mg/kg morphine (session × treatment: *F* (7, 62) = 34, *p* < 0.0001; Sidak tests: *p* < 0.0001), but not in rats receiving 1 or 3 mg/kg morphine (*p* > 0.19 and *p* > 0.12, respectively; Figure 3(f)). The carrageenan effect was significantly higher in the vehicle group compared to the 1 mg/kg group, but was similar for the 1 and 3 mg/kg groups (one-way ANOVA: *F* (3, 31) = 34.1, *p* < 0.0001; 1 mg/kg versus vehicle: *p* < 0.0001; 3 mg/kg vs 1 mg/kg: *p* = 0.92; Figure 3(g)).

Thus, the 1 mg/kg dose was sufficient for achieving the maximal analgesic effect of morphine in the HET and RTPA paradigms, but not in the Hargreaves test. Given the stronger efficacy of opioids in alleviating the aversive aspect of pain compared to its sensorimotor aspect, these results further support the notion that the HET paradigm, similarly to the RTPA paradigm, can be used to assess pain aversion.

### Blocking excitatory transmission at the lateral parabrachial nucleus similarly abolishes carrageenan-induced heat hypersensitivity as measured by the Hargreaves and HET paradigms

**Figure 4.**
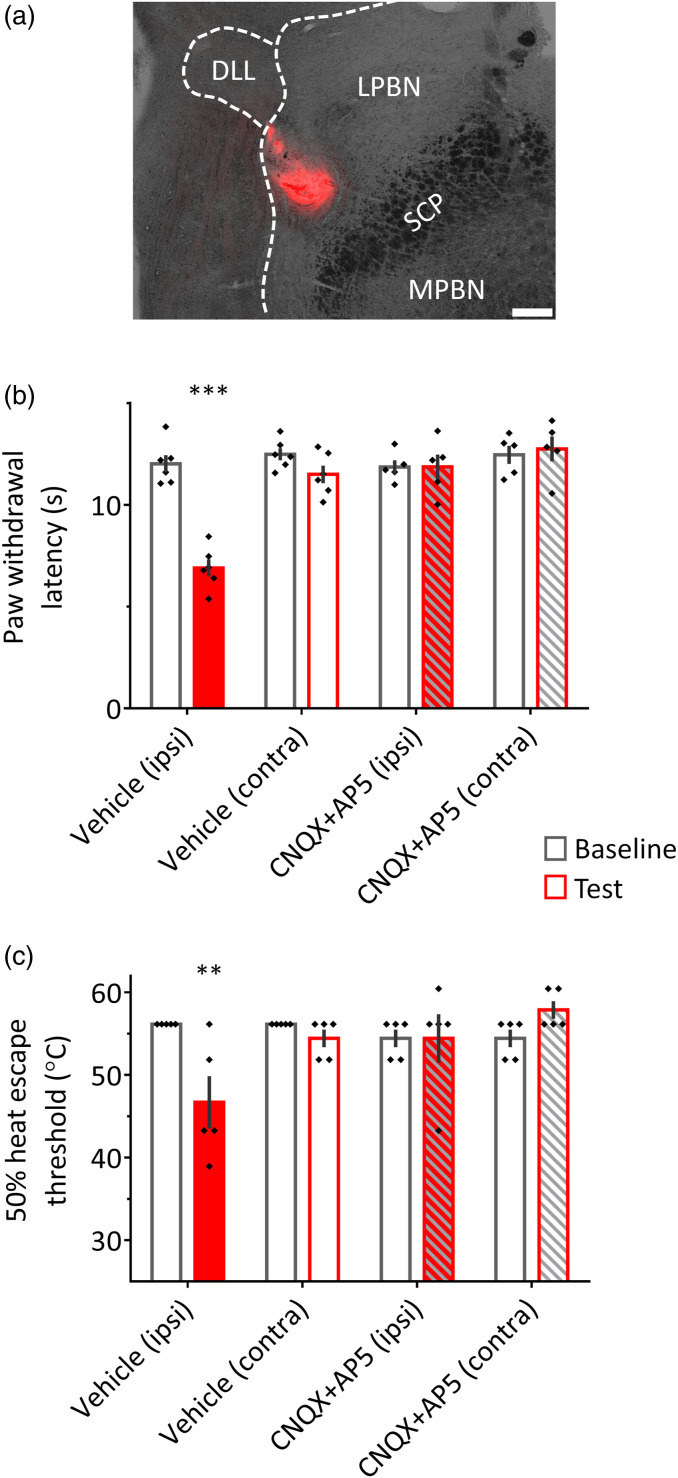
Blocking of glutamatergic input to the lateral parabrachial nucleus abolished carrageenan-induced hypersensitivity to heat as measured by both the Hargreaves and HET tests. (a) A typical example of an injection location within the LPBN, marked by DiD (red). DLL: Dorsal nucleus of the lateral lemniscus; LPBN: lateral parabrachial nucleus; MPBN: Medial parabrachial nucleus; SCP: Superior cerebellar peduncle. (b) In the Hargreaves test, carrageenan treatment caused a reduction in paw withdrawal latencies in animals receiving an intra-LPBN microinjection of vehicle (*n* = 6), but not CNQX + AP5 (*n* = 5). (c) In the HET test, rats receiving vehicle injection in the LPBN showed a significant reduction in escape thresholds for the carrageenan-treated paw. In contrast, no reduction in escape thresholds was observed in animals receiving intra-LPBN CNQX + AP5. *n* = 5 per group. **p < 0.01, ***p < 0.0001 compared to the baseline session, and compared to the control hindpaw in the test session. “Ipsi” and “contra” refer to carrageenan-treated and control hindpaws, respectively. Filled red bars indicate carrageenan treatment, and diagonal lines indicate intra-LPBN CNQX + AP5.

We and others have recently shown that inhibition of LPBN neurons inhibits paw withdrawal in response to radiant heat.^22,36^ Here, we aimed to examine whether blocking of excitatory input to the LPBN would similarly affect carrageenan-induced hypersensitivity as measured by the Hargreaves and HET paradigms. For both paradigms, rats were tested before and after carrageenan treatment of the left hindpaw (baseline and test sessions, respectively). Prior to the test session, rats received bilateral intra-LPBN microinjections of either glutamate antagonists (CNQX + AP5) or vehicle (Figure 4(a)).

The results of the Hargreaves test are shown in Figure 4(b). In rats receiving intra-LPBN injections of vehicle, paw withdrawal latencies were significantly lowered following carrageenan treatment compared to baseline (6.9 ± 0.4 s and 12 ± 0.4 s, respectively), and compared to the control hindpaw in the test session (11.5 ± 0.4 s; session x treatment: *F* (3, 18) = 24.4, *p* < 0.0001; Sidak tests: *p* < 0.0001). In contrast, carrageenan-induced hypersensitivity was not present following intra-LPBN injection of glutamate antagonists (*p* > 0.7). No differences between baseline and test sessions were detected for the control hindpaw in rats receiving intra-LPBN vehicle or glutamate antagonists (*p* = 0.22 and *p* > 0.97, respectively).

Similar results were observed for the HET paradigm (Figure 4(c)). In rats receiving intra-LPBN vehicle injections, carrageenan treatment effectively reduced the 50% HET as compared to baseline (46.7 ± 3.2°C and 56.2 ± 0°C, respectively), and compared to the control hindpaw in the test session (54.4 ± 1.1°C; session x treatment: *F* (3, 16) = 6.2, *p* = 0.005; Sidak tests: *p* < 0.009). In contrast, carrageenan treatment failed to reduce the 50% HET in rats receiving intra-LPBN injections of glutamate antagonists (*p* > 0.65). For the control hindpaw, there was no significant difference between baseline and test sessions (vehicle: *p* > 0.9; CNQX + AP5: *p* > 0.44).

Thus, pharmacologically blocking glutamatergic inputs to the LPBN similarly reversed carrageenan-induced changes in reflexive withdrawal and escape, as measured by the Hargreaves and HET tests, respectively. These results are consistent with the notion that the LPBN plays an important role in both the sensorimotor and emotional aspects of pain behaviour.^36,37^

### Acute opioid withdrawal induces heat aversion as measured by the HET paradigm without affecting reflexive responses to radiant heat

**Figure 5.**
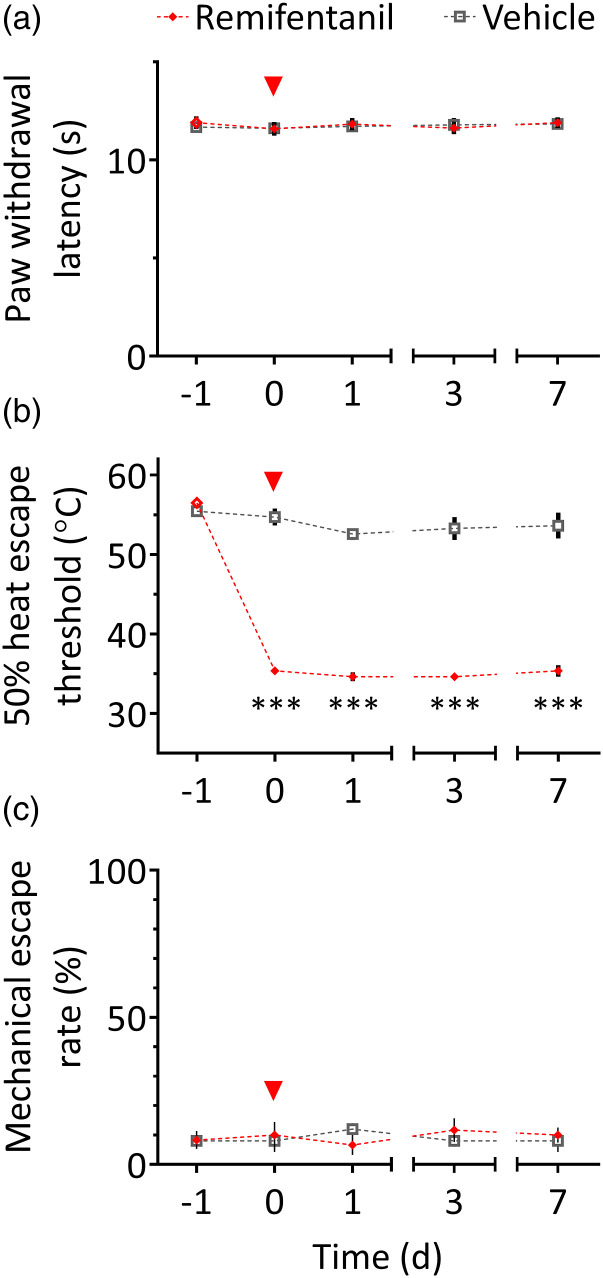
Acute opioid withdrawal does not affect paw withdrawal responses to heat, but causes a strong reduction in heat escape thresholds. Baseline measurements were performed on day −1; on day 0 (4 h before testing), rats received an acute intravenous infusion of either remifentanil or vehicle (saline) for one hour, under isoflurane anaesthesia (red arrowhead). For each rat, measurements from both hindpaws were averaged. (a) No effect of remifentanil withdrawal was observed in the Hargreaves test (*n* = 6 per group). (b) In the HET paradigm, remifentanil-treated rats showed markedly reduced escape thresholds starting from day 0 and until the end of testing (*n* = 6 per group). (c) Stimulation with an unheated probe rarely elicited escape responses in either vehicle-treated (8.8 ± 1.2% of trials, *n* = 5) or remifentanil-treated rats (9.3 ± 2.6% of trials, *n* = 6). ***p < 0.0001 compared to day −1, and compared to the vehicle group on the same day.

We next sought to examine the ability of the HET paradigm to detect increased heat aversion in an animal model of generalized hyperalgesia. For this, we chose an acute remifentanil withdrawal model, which causes long-lasting mechanical hypersensitivity as measured with the von Frey test,^24^ but does not affect responses to radiant heat in the Hargreaves test (unpublished data). As expected, remifentanil withdrawal did not induce a change in paw withdrawal latencies in the Hargreaves test (session x treatment: F (4, 40) = 0.2, *p* = 0.92; Figure 5(a)). In contrast, in the HET paradigm (Figure 5(b)), remifentanil-treated rats exhibited reduced escape thresholds in all test sessions (35 ± 0.4°C, on average) compared to the baseline session (56.5 ± 0.4°C), and compared to control animals in test sessions (53.6 ± 1.2°C, on average; session × treatment: *F* (4, 40) = 63.2, *p* < 0.0001; Sidak tests: *p* < 0.0001). To exclude the possibility that the difference between the Hargreaves and HET results is related to withdrawal-induced mechanical hypersensitivity, the HET experiment was repeated with an unheated probe (Figure 5(c)). Here, no significant session × treatment interaction was detected (*F* (4, 36) = 0.8, *p* = 0.56); overall, escape responses were observed in 9.1 ± 1.4% of trials. These results suggest that withdrawal from a single high dose of remifentanil induces heat aversion without affecting discrete nocifensive responses to heat.

## Discussion

The measurement of discrete reflexive responses to potentially painful stimuli has been the gold standard in preclinical pain research for many decades, and has substantially contributed to our mechanistic understanding of pain processing. Nonetheless, the relevance of these methods to the development of treatments for human pain patients has been questioned in recent years.^2,8–10^ In particular, such tests do not measure the aversive aspect of pain, which constitutes a major burden faced by pain patients. In response to this criticism, methods aimed at examining the emotional-motivational component of pain have become increasingly common in preclinical animal studies.^10,28,38–40^

Since pain is a strong innate motivator, one approach to study the perceived aversiveness of somatosensory stimuli in animal subjects is to examine the ability of these stimuli to act as teaching signals in aversive learning paradigms. For example, the involvement of the LPBN and amygdala in processing pain aversion has been demonstrated in studies employing classical threat conditioning, in which a previously neutral signal (e.g., tone) is experienced as threatening following its pairing with a noxious shock.^27,36,41–43^ However, while noxious electrical stimulation is extremely effective as an aversive teaching signal, it is a temporally restricted and unnatural stimulus, and as such cannot be considered a clinically relevant pain model. Therefore, many studies utilise an alternative conditioning paradigm, namely CPP/CPA, in which a specific location within a behavioural arena is paired with the presence of pain (for CPA), or a pain-relieving intervention (CPP). Achieving robust and reproducible CPP/CPA in rodents is technically difficult, typically requires multiple acquisition sessions, limited to short-lived pain models or pain relief, and relies on long-term memory,^12–16,18^ complicating interpretation. As a partial solution, some studies have utilised RTPA paradigms (sometimes also referred to as place escape/avoidance paradigms), in which animals are driven to occupy a specific location in order to escape and avoid noxious or aversive input in real-time.^20–23^ In the context of pain research, such paradigms typically employ localized pain models to allow the same somatosensory stimulation to be experienced as neutral when applied to one (untreated) hindpaw but as painful when applied to the contralateral hindpaw (e.g., following carrageenan treatment, see Figure 2(f)). While not requiring long-term learning, such paradigms undoubtedly involve a cognitive component, as the animal must comprehend the relationship between its location in space and the aversiveness of the administered stimulation, and choose an active avoidance strategy (as opposed to freezing, for example). In addition, such a protocol cannot be used to detect pain aversion in animal models of generalized pain, such as opioid withdrawal-induced hyperalgesia or chemotherapy-induced pain.^10,24–26,44^

To circumvent these issues, some studies have utilised unconditioned behaviour. For example, thermal gradient tracks can be used to examine the animal’s preference to specific temperature ranges.^28,45^ However, the detection of thermal hyperalgesia in localized pain models (e.g., intraplantar carrageenan) is likely to be at least partially masked by paw guarding behaviour, which allows the animal to occupy warm regions of the gradient while avoiding direct contact of the affected paw with the heated surface. This problem would be especially pronounced in larger animals such as adult rats, where the distance between the lifted paw and the surface can be substantial. In recent years, stimulus-evoked escape behaviour has been suggested as a measure of pain aversion in rodents. In particular, jumping behaviour in mice has been instrumental in uncovering neuronal mechanisms underlying the emotional-motivational component of pain.^23,27,28^ In contrast, despite the widespread use of rats in preclinical pain studies, there is currently no prevalent method for the evaluation of pain-evoked escape behaviour in this species, which does not typically exhibit jumping behaviour (at least in adulthood). We propose that this gap could now be filled by the HET paradigm.

Heat hyperalgesia is a hallmark of numerous pain pathologies of various aetiologies, including inflammatory and neuropathic pain.^46,47^ Our data demonstrate that the HET constitutes a robust and reproducible method for assessing heat pain aversion in both male and female rats, and across experimenters and pain models. A temperature that reliably evoked escape in inflamed paws in the HET paradigm could also induce RTPA in carrageenan-treated rats. In inflammatory pain rats, systemic administration of 1 mg/kg morphine was sufficient for blocking the carrageenan effect in the HET and RTPA paradigms, but not in the Hargreaves test. This is in line with previous studies showing a more potent action of opioids on the aversive compared to the sensorimotor aspect of pain.^13,16,18,34,35^ However, to the best of our knowledge, the current study is the first to report such findings in the context of heat pain in rodents. Importantly, the reduced escape observed in morphine-treated rats was not caused by a general decrease in locomotion (see Figure 3(d)).

We propose the HET paradigm as a powerful tool for studying the mechanisms underlying the aversive aspect of heat hypersensitivity and its modulation in pathological states, which are currently not well understood. For example, we have demonstrated a dissociation between reflexive and aversive responses to heat following withdrawal from a single high dose of remifentanil (Figure 5). We have previously shown that this treatment leads to mechanical hypersensitivity in rats.^24^ In contrast, thermal hypersensitivity in this model could not be detected using the Hargreaves test (Figure 5(a)). These results could lead to the conclusion that withdrawal from acute remifentanil treatment induces plasticity in pathways related to mechanical, but not thermal, somatosensation. The HET data do not support this conclusion, but rather suggest that heat aversion is more strongly affected by remifentanil withdrawal than reflexive responses to heat. Additional work would be required to elucidate the physiological mechanisms underlying this dissociation, and its generalization to other opioids. One intriguing possibility is that the greater efficacy of opioid treatment and opioid withdrawal in alleviating and inducing pain aversion, respectively, share common mechanisms.

We show, for the first time, that the blocking of excitatory input to the LPBN abolishes the effects of carrageenan on both reflexive and aversive responses to heat. This result is in agreement with existing literature, showing that the LPBN is involved in multiple aspects of pain.^36,37,48–50^ Interestingly, we observed that disruption of LPBN excitation reversed carrageenan-induced behaviour, but did not affect responses to stimulation of the untreated paw (Figure 4). This is in contrast to previous studies, where LPBN inhibition led to reduced sensitivity to radiant heat both in the presence and absence of a pain model.^22,36^ Thus, it seems that the effects of LPBN inhibition on pain behaviour are not merely the result of antagonising sensory-evoked excitatory input to the LPBN, but rather reflect a reduction of LPBN activity compared to baseline levels. How different LPBN circuits control various aspects of pain behaviour is only beginning to be revealed.^23,37,51^ Future studies could utilise the HET paradigm to target LPBN circuits involved in aversive responses to heat.

The current study focused on quantifying escape responses evoked by heat stimulation. It would be intriguing to examine whether escape thresholds to other types of somatosensory stimuli could be used in a similar way. Cold allodynia is a common symptom of several pain pathologies, including neuropathies following injury and chemotherapy.^46,52^ Future thermal probes could employ the Peltier effect to deliver controlled cold stimulation, which could potentially be used to quantify cold escape thresholds in relevant rodent pain models such as chronic nerve constriction or systemic oxaliplatin.^10,25^ Similarly, mechanical allodynia is prevalent in many pain conditions.^46,47^ Von Frey filaments are most often used to quantify paw withdrawal thresholds to mechanical stimulation, but high-force filaments can induce escape in pain model rats.^20,21^ Potentially, escape thresholds to mechanical stimulation could be quantified using a similar protocol as the HET paradigm.

Recent years have seen a rapid expansion of methods utilised in the assessment of pain-related behaviour, leading to a deeper understanding of pain physiology and pathology.^9,11,53^ Based on the results obtained in the current study, we propose the HET paradigm as a powerful, practical, and cost-effective addition to the toolbox of behavioural preclinical pain researchers. In combination with other methods, the HET can facilitate the dissection of neuronal populations and pathways underlying the various aspects of pain.
